# Similar outcomes after anterior cruciate ligament reconstruction in paediatric and adult populations: a 1-year follow-up of 506 paediatric operations in Denmark

**DOI:** 10.1007/s00167-023-07530-9

**Published:** 2023-08-12

**Authors:** Maria Østergaard Madsen, Susan Warming, Martin Wyman Rathcke, Peter Faunø, Torsten Grønbech Nielsen, Robert Bennike Herzog, Mathilde Lundgaard-Nielsen, Anette Holm Kourakis, Martin Lind, Michael Rindom Krogsgaard

**Affiliations:** 1 Section for Sports Traumatology M51, Bispebjerg and Frederiksberg University Hospital, (A Part of Copenhagen IOC Research Center), Bispebjerg Bakke 23, 2400 Copenhagen, NV Denmark; 2 Department of Physical and Occupational Therapy, Bispebjerg and Frederiksberg University Hospital, Copenhagen, Denmark; 3 Sector for Sports Traumatology, Aarhus University Hospital Skejby, Aarhus, Denmark

**Keywords:** Paediatric, Knee, ACL, Injury, Reconstruction, Re-rupture

## Abstract

**Purpose:**

To present 1-year results after all paediatric anterior cruciate ligament (ACL) reconstructions in Denmark (5.9 M inhabitants) for the 10½ year period, 1 July 2011 to 31 December 2021.

**Methods:**

All children who had an ACL reconstruction were enrolled. They were asked to complete Pedi-IKDC preoperatively and at 1-year follow-up. Independent observers performed pivot shift test and instrumented laxity assessment preoperatively and at 1-year follow-up.

**Results:**

The median age of the 506 children (47.2% girls) was 14.3 years (9.3–15.9). The Pedi-IKDC score increased from preoperatively 61.6 ± 15.8 (mean ± SD) to 85.9 ± 13.0 at 1-year follow-up (*p* < 0.0001). There were concomitant injuries (to meniscus and/or cartilage) in 49.9%, but these children had preoperative and follow-up Pedi-IKDC scores similar to the scores for children with isolated injury to ACL (n. s.). Instrumented anterior laxity was 4.3 ± 1.4 (mean ± SD) mm preoperatively and 1.4 ± 1.4 mm at follow-up (*p* < 0.0001). Preoperatively, 3% had no pivot shift whilst this was the case for 68% postoperatively (*p* < 0.0001). Twenty-five children (5.6%) had 4 mm instrumented laxity or more relative to the unoperated knee at follow-up. Two patients (0.4%) had an operatively treated deep infection, three (0.5%) were operated on for reduced range of motion and two (0.4%) had a revision ACL reconstruction.

**Conclusion:**

ACL reconstruction resulted in a clinically meaningful increase in Pedi-IKDC, an improved instrumented stability, a reduction in the grade of pivot shift and the complication rate was low at 1-year follow-up. The risk of graft insufficiency at 1-year follow-up was the same as in an adult population.

**Level of Evidence:**

II.

**Supplementary Information:**

The online version contains supplementary material available at 10.1007/s00167-023-07530-9.

## Introduction

Knee ligament injuries are not common in children and the treatment strategy is debated. The largest treatment series to date include 237 children [17], and there is the only one other series with more than 100 children. Only 15 of 58 series in literature include more than 50 children (supplementary Table S1).

Based on data from the Danish Knee Ligament Reconstruction Register, there is a high risk for revision surgery after ACL reconstruction (ACL-R) in Danish children and adolescents compared to adults [5], even though the reason for this is not fully understood. In addition to technical failure with recurrent laxity in children, an explanation for the high revision rate can be that children more often than adults return to high risk activities. However, this is hypothetical.

The treatment strategy of paediatric ACL insufficiency is debated [19]. There is no randomised controlled study to back-up either of the two major strategies: reconstruction in all children with ACL injury, or rehabilitation and reconstruction when needed. There are good results of a non-surgical strategy in Scandinavian children with 33% needing surgery within a mean observational period of 3.8 years [20], 55% at a mean of 8 years following injury [2] and 57% after a mean of 9.5 years [3]. In the combined group of operated and non-operated children, 34% had a meniscal injury within a mean of 9.5 years [3]. Secondary reconstruction in other series with rehabilitation as primary treatment is reported in 58% after 70 months [21] and 68% after 3–5 years [15]. In a retrospective study of 56 ACL-reconstructed children from 2 hospitals with different treatment strategies, those who had a reconstruction early after injury showed better results than children who were operated later, as evaluated with the International Knee Documentation Committee Subjective Knee Evaluation Form (IKDC-SKF) [11] and similar results were reported in a series of 31 children [23] in which children with coexisting injury to meniscus or cartilage had ACL-R whilst children with isolated ACL injury had surgery if needed. Based on a meta-analysis of series with a total of 1176 children—463 primarily operated, 459 treated with delayed operation and 254 unoperated—early reconstruction was recommended to secure the best outcome [14]. However, there are no randomised, controlled studies, and there is a risk of selection bias in small series.

The purpose of the current prospective study was to report the 1-year outcome in a large, unselected group of consecutive children who had an ACL-R, covering all cases in Denmark.

## Materials and methods

Data were registered according to a permission from the Danish Data Protection Agency (jr. nr. 2012-58-0004). The ethical committee for the Capital Region stated that a specific permission was not necessary, as there was no intervention involved (H-20050959).

**Fig. 1:**
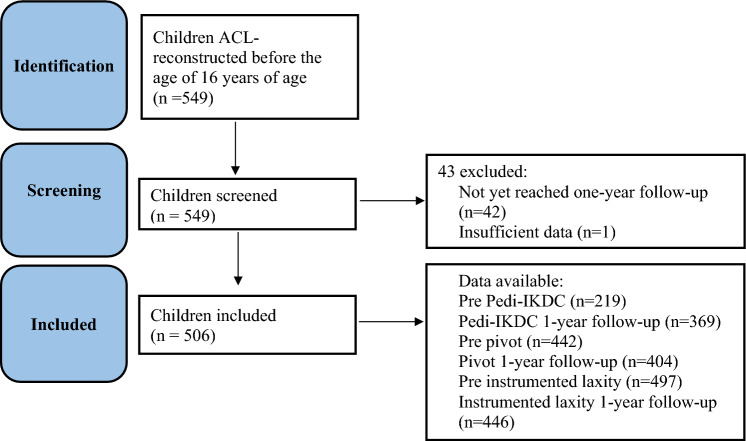
Flowchart showing the included series of children and available data

Since July 1, 2011, the surgical treatment of children (defined by the Danish Board of Health as persons < 16 years of age) with anterior cruciate ligament injury in Denmark (5.932 M inhabitants per 1 January 2023) has been centred at two public hospitals. The same five orthopaedic surgeons have performed these operations since 2011, three in centre 1 and two in centre 2. The material consisted of all children who had an ACL-R before the age of 16 (Fig. 1). Baseline data including operative details and information about concomitant knee injuries have been prospectively registered. Children have been followed prospectively—at centre 1 regularly until the age of 18 years, and at centre 2 until 1 year after reconstruction. Children who had an ACL-R have been evaluated before surgery and at 1-year follow-up by Pedi-IKDC, clinical knee stability measures and instrumented knee laxity measures. Complications and additional treatments were registered at follow-up. To reduce the risk of bias, physiotherapists and not the operating surgeons generated the follow-up data in both centres. There were five physiotherapists and all were experienced with knee testing before involvement in the study and have been part of the study for 8–10.5 years. The current study is based on data from all children treated with ACL-R at the two clinics during the 10½ year period, 1 July 2011–31 December 2021, and results from 1-year follow-up.

### Surgical procedure

All children with persisting epiphyseal growth plates were operated using a paediatric technique to avoid physeal arrest (as described in the supplementary material). For patients with closed growth plates, a standard adult technique was used, with anatomic positioning of the femoral tunnel by anteromedial technique. Postoperative full weight bearing was allowed. In one centre, the newly implanted ACL graft was protected in a brace with flexion limited to 40° for 4 weeks. Rehabilitation was supervised by physiotherapists. The rehabilitation programme was the same in both centres and is described in the supplementary material.

### Stability measurements

Clinical stability was assessed by the pivot test and valgus/varus stress tests. Instrumented laxity at 30° knee flexion was measured using the Rolimeter®, as described by Ganko et al. [8]. Pivot shift was graded 0 (absent), grade I (slight), grade II (definite subluxation), and grade III (subluxation and momentary locking) [1].

### Patient-reported outcome measure (Pedi-IKDC)

Pedi-IKDC was developed from the adult 19-item IKDC-SKF by asking 30 children about their understanding of the 19 items [12]. IKDC-SKF has inadequate construct validity for adults [9], and there is no certainty that Pedi-IKDC has content validity for children. Construct validation of Pedi-IKDC has shown that for children with ACL injury, the unidimensional questionnaire does not exhibit adequate fit, meaning that measurement properties are inadequate, and it is recommended that data obtained by Pedi-IKDC are interpreted with caution [10]. The Danish version was translated by the forward-backward method, culturally adapted through interviews with 45 children, and validated by classical test method analyses [13]. Responsiveness was found to be good in children treated for ACL injury.

### Statistical analyses

All descriptive analyses were performed using SPSS (IBM SPSS Statistics for Windows, Version 25. Armonk, NY: IBM Corp). Missing data were regarded as randomly distributed and were not compensated for in the statistical analyses. For Pedi-IKDC and the clinical measurements, mean differences between pre-measurement and 1-year follow-up were calculated and analysed by paired samples *T* test. For the Pedi-IKDC, the aggregated score was calculated as percentage of the maximum possible score, according to the scoring instructions.

Results are reported as mean (± SD) as well as median (range), because data are not normally distributed. Differences in the scores of Pedi-IKDC between the groups with and without concomitant injury were analysed by independent *T* test samples. *p* value of significance was 0.05. Correlations were calculated as the Pearson coefficient.

For comparison of Pedi-IKDC scores for patients with and without concomitant injuries (to meniscus or cartilage), 35 patients are necessary in each group to be able to show a difference in aggregated score of 10% points, provided that SD is 15% points, alpha is 0.05 and beta is 0.8.

## Results

Figure 1 describes the cohort. The baseline data for the 549 children, who were operated at the two departments with an ACL-R during the 10½ year period, are listed in Table 1.

**Table 1:** Baseline values for 549 children who had an ACL reconstruction during a 10½ year period in Denmark

ACL-reconstructed children as of December 31, 2021	549
Boys/girls (*n* (%))	290 (52.8)/259 (47.2)
Mean age (SD) (range)	14.1 (± 1.3) (9.3–15.9)
Injured knee: right/left (*n* (%)) (*n* = 548)	254 (46.3)/294 (53.6)
Mean time between injury and operation (SD) (range) (*n* = 535)	6 months (± 8 months), (0–127 months)
Open epiphyses/closed epiphyses at the time of operation (*n* (%)) (*n* = 517)	463 (84.3)/54 (9.8)

Injuries to meniscus and cartilage were confirmed during the arthroscopic examination in connection with ACL-R. There were 87 children with a medial and 151 with a lateral meniscal injury (corresponding to 16% and 27.5% of all children, respectively), and 15 children (3%) had a lateral as well as a medial meniscal lesion. There were 26 (5%) with cartilage lesions to the medial and 14 (3%) to the lateral femoral condyle, 6 to the medial and 3 to the lateral tibial plateau, 8 to patella and 6 with undescribed location. The concomitant injuries are listed in Table 2. A detailed description of the treatment of meniscal injuries is presented in Table 3.

**Table 2:** Concomitant injuries in the cohort of ACL-reconstructed children (*n* = 549) during a 10½ year period in Denmark

Meniscal injury	193 (35.2%)
Cartilage injury	41 (7.5%)
Meniscal + cartilage injury	26 (4.7%)
Medial collateral ligament injury	8 (1.5%)
Meniscal + medial collateral ligament injury	4 (0.7%)
PCL injury	1 (0.2%)
Femoral avulsion of ACL	1 (0.2%)

A total of 274 children (49.9%) had concomitant injuries. Children are only counted once in the table

**Table 3:** Treatment of meniscal injuries in 223 ACL-reconstructed children

Treatment	Medial meniscus (*n* = 87)	Lateral meniscus (*n* = 141)
Reinsertion (suture)	52 (60%)	62 (44%)
Resection	18 (21%)	46 (33%)
Root insertion	2 (2%)	6 (4%)
No treatment	13 (15%)	30 (21%)
No data	2 (2%)	7 (5%)

Fifteen children had lesions in both menisci and are counted twice. Percentage refers to medial and lateral meniscus, respectively

The graft diameter was mean (± SD) 8.2 mm (± 0.8) with a median of 8.0 mm and range of 5–10 mm. The grafts that were used are listed in Table 4.

**Table 4:** The grafts used for the reconstruction of ACL in 549 children

Four-doubled semitendinosus	415 (75.6%)
Three-doubled semitendinosus + doubled gracilis	4 (0.8%)
Three-doubled semitendinosus + 3-doubled gracilis	1 (0.2%)
Doubled semitendinosus + doubled gracilis	96 (17.5%)
Gracilis, no specification	3 (0.5%)
Allograft	5 (0.9%)
Quadriceps tendon-bone	13 (2.4%)
No information	6 (1.1%)
Total	549 (100%)

All except five were autografts

The mean increase of 24.3 points (median 27.7 points) in preoperative to 1-year follow-up scores was significant (*p* < 0.0001). There was no significant difference between the scores (preoperatively and at 1-year follow-up) from patients with concomitant injuries and patients without concomitant injury (n. s.).

There was significant improvement from preoperative scores to 1-year follow-up scores in all groups (*p* < 0.0001). Preoperative and follow-up Pedi-IKDC scores are presented in Table 5.

**Table 5:** Pedi-IKDC scores (% of maximum score) preoperatively and 1-year postoperatively for the cohort of 506 paediatric ACL-reconstructed patients

Preoperatively	Pedi-IKDC score	Postoperatively	Pedi-IKDC score
All patients (*n* = 219)		All patients (*n* = 369)	
Mean* (*SD)	61.6 (± 15.8)	Mean* (*SD)	85.9 (± 13.0)
Median (range)	62.5 (9.8–97.8)	Median (range)	90.2 (12–100)

Patients with concomitant injury (*n* = 86)		Patients with concomitant injury (*n* = 143)	
Mean (SD)	60.4 (± 16.4)	Mean (SD)	85.3 (± 14.6)
Median (range)	62.0 (9.8–89.1)	Median (range)	85.3 (12–100)

Patients without concomitant injury (*n* = 133)		Patients without concomitant injury (*n* = 226)	
Mean (SD)	62.0 (± 15.4)	Mean (SD)	86.2 (± 11.9)
Median (range)	63.0 (17.4–97.8)	Median (range)	90.2 (41.3–100)

The mean instrumented anterior side-to-side laxity measurement was 4.3 mm (SD ± 1.4) preoperatively and 1.4 mm (SD ± 1.4) at follow-up (*p* < 0.0001). The mean reduction in preoperative laxity to 1-year follow-up (in the individual patient) was 3.0 (SD ± 1.8). The distribution of laxity at 1-year follow was: < 2 mm: 83.4%, 2½–5 mm: 14.8% and > 5 mm: 1.8%. Twenty-five children (5.6%) had a laxity of 4 mm or more. There was a significant (*p* = 0.003) but weak correlation (Pearson coefficient: 0.146) between graft diameter and laxity at 1-year follow-up.

**Fig. 2:**
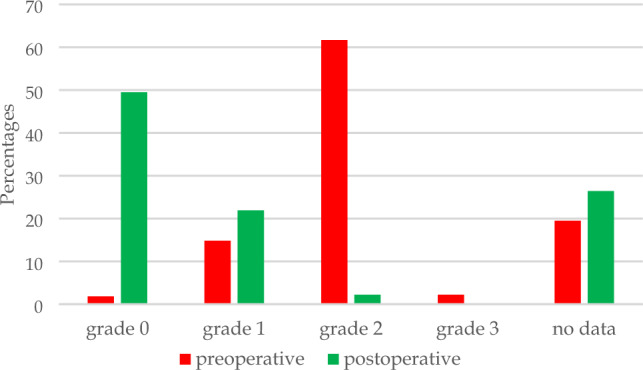
Pivot shift preoperatively (*n* = 503) and at 1-year follow-up (*n* = 453) (*p* < 0.0001)

Figure 2 shows the changes in the pivot shift from preoperatively to 1-year follow-up. The mean difference in grade was 1.6 (SD ± 2.7) (*p* < 0.0001). Sixty-eight per cent of the children for whom information about pivot shift was available at follow-up had no pivot, and 96% had grade A (equivalent to no pivot + grade 1).

There were five complications: two children (0.4%) had an operatively treated deep infection and three (0.5%) were treated for reduced range of motion. Two children (0.4%) had revision ACL-R within the first year: one 10 months after primary surgery after a trauma during football, and the other 12 months post-surgery without any new trauma. The diameter of the original ACL graft in the two cases who had a revision ACL-R was 8 and 9 mm, respectively.

## Discussion

The main findings of the study were that paediatric ACL-R resulted in a substantial increase in mean Pedi-IKDC score and a reduction of instrumented laxity and pivot shift. The results of this study indicate that the subjective and clinical outcomes 1 year after ACL reconstruction are comparable to results from adult populations.

The complications after paediatric ACL-R have been studied in large treatment databases. In a register analysis of more than 16,000 procedures in children and adolescents (< 20 years of age) in UK, the rate of deep infection was low (0.31%) and the risk of re-rupture was 7.5% after 5 years (and 6.37% for ACL injury in the contralateral knee) [22]. In a report from the Swedish National Knee Ligament Register of 569 reconstructions in persons < 20 years of age, the 5-year revision rate was 3.9–6.9%, with the highest figure in the youngest segment of the cohort [24]. Data from the Danish Knee Ligament Reconstruction Register show that for children who were 13–15 years at the time of ACL reconstruction, the 2-year revision rate was 4.8% and the 5-year revision rate was 10.4% [5]. The main reasons for revision surgery was a new trauma in 59% and suboptimal graft placement in 27% [5]. For children who were < 13 years old at the time of reconstruction, the revision rates were 1.3% (2 years) and 7.4% (5 years), respectively [5]. However, the clinical and subjective outcomes were not described in detail in these epidemiological studies [5, 22, 24], and failure and re-rupture rates are probably underestimated, as they are only expressed as re-operated cases. The instrumented laxity at 1-year follow-up for all age groups in the Danish Knee Ligament Reconstruction Register was ≤ 2 mm: 84.6%, 3–5 mm: 13.7% and > 5 mm: 1.7% [6], and this is comparable to figures in the current paediatric cohort. This indicates that the technical result (in particular stability) after ACL-R is the same in children and adults, and that the higher revision rate in children may reflect higher activity and stronger desire to return to pivoting sports and perhaps lack of self-recognition of the limitations in relation to knee function.

It is a general concern that the revision rates after paediatric ACL-R are high [5], in particular in boys. The reported long-term rates of 11–28% [21, 25] are twice as high as the revision rate in young adults (20–35 years of age) [23]. Such high revision rates could not be confirmed in the present study, in which only two (0.4%) were revised, but the follow-up period is short, and only about half of the re-ruptures usually occur during the first year after reconstruction [25]. However, in literature, the long-term re-rupture rate seems quite similar to the risk of rupturing the contralateral ACL [21, 22, 25], and in a study with a mean follow-up of 4 years, the re-rupture rate was 12% whilst the contralateral rupture rate was 16% [16]—supporting the view that re-rupture of a reconstructed ACL is mainly related to behaviour of the patient and to a lesser degree to insufficiency of the reconstruction and rehabilitation. In line with this, a recent meta-analysis of the risk for contralateral ACL rupture concluded that return to high-level sports is the most important factor for injury [4]. Activity level was not registered at 1-year follow-up in this cohort, but the majority of children had most likely not returned to previous activities. Many children (and their parents) used the 1-year follow-up as an opportunity to be cleared for returning to sports. If technical insufficiency of the reconstruction was a main reason for re-rupture, it would be expected to occur shortly after return to activity. However, a more gradual increase in re-rupture rate is reported [5], which indicates that the factors for re-injury are cumulative. An important indicator could be cumulated hours of sports activity.

The vast majority of previous series on the outcome of paediatric ACL-R use PROMs that have been developed for adults [14]. It is well described that IKDC and KOOS are not meaningful for children [12, 26], and the results obtained with such PROMs from children can at best be regarded as a brief indication of the outcome. Pedi-IKDC and KOOS-Child are modified versions of IKDC and KOOS, and some of the difficulties for children in relation to the understanding of items have been compensated for. However, there is little confidence to the content validity of IKDC and KOOS for adults with ACL injury [9, 10, 12, 13], and, therefore, the content validity of Pedi-IKDC and KOOS-Child for children with ACL injury is questionable. The finding of a large increase in scores of Pedi-IKDC in the present study after ACL reconstruction can, therefore, be regarded as indication of a much better knee function in general and not necessarily a measure specifically related to the problems after ACL injury. Even though it is unspecific, it indicates good outcome after the treatment in the present cohort. The finding that there is no difference between Pedi-IKDC scores from children with isolated injury to ACL and children with additional injuries (in particular meniscal and cartilage injury) can be true, or it can represent a type-2 error, introduced because Pedi-IKDC is not specific and sensitive enough to measure such a difference.

The current study of 1-year results following paediatric ACL-R and rehabilitation is relevant in relation to assessment of the surgical procedure and rehabilitation. It can be subject for discussion how a failed reconstruction is defined. Re-rupture is in most cases a result of a new trauma, whereas insufficiency of the ACL-R can be defined as > 2 mm side-to-side difference, > 4 mm difference or the presence of a pivot shift. No matter how failure is defined, the current study shows that ACL reconstruction is technically successful to the same degree in children and in adults within the first year after surgical treatment [6].

It is a strength that the study covers all paediatric ACL reconstructions in a whole country and that there is no selection bias. The follow-up period of 1 year is short, but long enough to describe the early complications in the form of infection, arthrofibrosis and cyclops formation. It is a limitation that preoperative and follow-up PROM data were only available for 43% and 73%, respectively, of the 506 children. However, the completeness of the 1-year follow-up data is higher than the response rate at follow-up in national quality databases, which is typically 29–35% [7, 18]. Of the 58 earlier series on outcomes after paediatric ACL-R (supplementary Table S1), only 11 reported preoperative as well as follow-up PROM data. Only three of these reported 100% completeness of PROM data. Amongst the 36 series that only reported follow-up data, 18 had 100% completeness (with a cohort of median 26 patients, range 10–94) and 7 had completeness between 39% and 82.5%. Based on literature, the completeness in relation to PROM data in the current study seems to be acceptable, although not optimal.

The short-term results of paediatric ACL-R in this large cohort show that technically this procedure leads to satisfactory outcomes. To reduce the high risk of re-rupture in children and adolescents, it, therefore, seems essential to focus on injury prevention, once they return to sports. Also, in light of the low incidence of paediatric ACL injury, this study shows that through centralization of the treatment at a national level, it is possible to create an unselected cohort of a size that is a reasonable basis for randomised, controlled studies—data from such studies are essential to increase the quality of treatment and to establish the best treatment algorithm for these children.

## Conclusion

Paediatric ACL reconstruction resulted in a substantial increase in Pedi-IKDC and a relevant reduction of instrumented laxity and pivot shift 1 year following surgical treatment. Complication rate was low and 1-year revision rate was 0.4%.

### Supplementary Information

Below is the link to the electronic supplementary material.

Supplementary file1 (PDF 58 KB)

Supplementary file2 (DOCX 31 KB)

Supplementary file3 (DOCX 62 KB)

## Data Availability

Anonymised data from the study are available from the corresponding author upon reasonable request.
