# Supplementary material for: Similar outcomes after anterior cruciate ligament reconstruction in paediatric and adult populations: a 1-year follow-up of 506 paediatric operations in Denmark
Source: Knee Surg Sports Traumatol Arthrosc. 2023 Aug 12;31(11):4871–7. doi: 10.1007/s00167-023-07530-9 (PMC10598128; doi:10.1007/s00167-023-07530-9)
Supplement: Supplementary file 1 — Supplementary file1 (PDF 58 KB) [file 167_2023_7530_MOESM1_ESM.pdf]

**Supplementary: General Rehabilitation procedure guidelines at the two centers in Denmark**

The first 4 weeks focus on extension/flexion of the reconstructed knee, reduce pain and swelling of the knee. Crutches are used in the beginning (approx. a fortnight). One center uses a brace for the first 4 weeks to normalize the daily activity level e.g., walking stairs and support the reconstructed knee.

At 4 weeks begin ergometer cycle with low load together with the focus on the ROM movement stability and strength training

At 8 weeks riding the bike outside

At 12 weeks swimming (crawl) and run a flight of stairs

At 16 weeks easy running on a straight surface

At 6 months breaststroke

At 10 months beginning at non-contact activity/sport e.g., badminton, tennis

At 12 months (at the earliest) begin contact sport
