# Supplementary material for: Similar outcomes after anterior cruciate ligament reconstruction in paediatric and adult populations: a 1-year follow-up of 506 paediatric operations in Denmark
Source: Knee Surg Sports Traumatol Arthrosc. 2023 Aug 12;31(11):4871–7. doi: 10.1007/s00167-023-07530-9 (PMC10598128; doi:10.1007/s00167-023-07530-9)
Supplement: Supplementary file 2 — Supplementary file2 (DOCX 31 KB) [file 167_2023_7530_MOESM2_ESM.docx]

**Paediatric ACL reconstruction in COUNTRY since July 1, 2011**

The country National Board of Health has defined the pediatric population as children < 16 years of age. Pediatric ACL reconstruction (ACLR) has been centralized at two hospitals in COUNTRY since July 1, 2011. The majority of children < 16 years of age are skeletally immature, but not all.

ACL reconstruction in skeletally immature patients is planned with a technique to minimize the effect of surgery on the growth plates. Therefore, boneplugs or fixation devices bridging the growth plates are not used, and transphyseal drilling is performed with low velocity drilling, avoiding sharp angles to reduce the growth plate drilling volume.

Before 2013 the tibial tunnel was transphyseal, positioned at the native insertion site of ACL, and the femoral tunnel was drilled by transtibial technique inside-out, leaving 2 mm of cortical bone posterolateral in the notch. The diameters of the drilled tunnels were equivalent to the graft diameter, within 0.5 mm increments. The graft was fixed using a rigid suspensory loop fixation (Endobutton TM – Smith-Nephew) on femur and a bicortical screw and washer (Spiked Washer TM – Smith-Nephew), positioned distal to the tibial epiphysis as tibial fixation. The graft was autologous doubled semitendinosus/gracilis tendon.

Since 2013 the femoral tunnel was positioned distal to the physis (guided by peroperative flouroscopy) at the native insertion site, leaving 2 mm of cortical bone posterolateral in the notch. The tibial tunnel was transphyseal, positioned at the native insertion site. Both tunnels were created with a retrodrill (in yyy: Twister®, Depuy and at xxxg: Flipcutter®, Arthrex), were 25 mm deep, and with a diameter adjusted to the size of the graft. The graft was secured using a flexible suspensory loop device at both ends (Tigth-rope®, Arthrex). The standard graft was autologous quadruple semitendinosus tendon, but depending on tendon dimensions gracilis tendon was added in some cases. The specific grafts are described in table xx.

Postoperative full weight bearing was allowed, and at xxx Hospital the newly implanted ACL-graft was protected in a brace with ROM -10 - 40° for 4 weeks.

In children with closed physes we used the same positioning of the graft, but tunnels on femur were drilled outside-in or inside-out, depending on surgeon’s choice. In most cases a suspensible loop fixation device (Tigth-rope® – Arthrex) was used for fixation. Postoperative full weight bearing was allowed and a brace was not used.
