# Supplementary material for: Similar outcomes after anterior cruciate ligament reconstruction in paediatric and adult populations: a 1-year follow-up of 506 paediatric operations in Denmark
Source: Knee Surg Sports Traumatol Arthrosc. 2023 Aug 12;31(11):4871–7. doi: 10.1007/s00167-023-07530-9 (PMC10598128; doi:10.1007/s00167-023-07530-9)
Supplement: Supplementary file 3 — Supplementary file3 (DOCX 62 KB) [file 167_2023_7530_MOESM3_ESM.docx]

| *Study* | *No. of patients* | *Treatment* | *Mean age at inclusion (years)* | *Mean follow-up time (years)* | *% failure (operatively treated re-rupture or revision)* | *PROM(s) used* | *% answers to PROMs* |
| --- | --- | --- | --- | --- | --- | --- | --- |
| Zhang (2023)[13] | 16 | P  - Robot  - Freehand | 12.2 (+1,3) | 2.6 (+0.38) | N | IKDC at follow-up | N |
| Kamei (2023)[4] | 10 | P | 12.1 (11-13) | 2,6 (1.0-7.5) | N | IKDC, KOOS, and Lysholm pre-op and at follow-up | N |
| Foissey (2022) [2] | 39 | T + P | 13.8 (+1.4) | 4.8 (3.5-6.2) | 1 (2.6%) | Pedi-IKDC and Lysholm at follow-up | N |
| Kew (2022)[5] | 100 | Not described | 16.1 (+1.4) | 4.0 (+1.2) | 12 (12%) re-ruptures | IKDC and KOOS at follow-up | N |
| Kitchen (2022)[6] | 40  40 | T  - No STR  - STR | 14.9 (9.3-18.8)  15.7 (9.5-18.7) | 2.4 (2-3)  2.3 (2-3) | 7 (17.5%)  5 (2%) | Lysholm at follow-up | 82.5 %  95 % |
| Shamrock (2022)[11] | 12 | P | 12.8 (+1.8) | 2.3 (1.1-5.2) | 2 (16.7%) | No PROMs | N |
| Maheshwer (2022) [8] | 59 | Not described | 16.0 (14.5-17) | 2 | Excluded | IKDC and KOOS at follow-up | 100 % |
| Zimmerer (2021) [56] | 22 | T | 13.1 | 17.4 | 3 (13.6%) re-ruptures | IKDC, KOOS and Lysholm at follow-up | N |
| Hansson (2021)[3] | 193 | T | 13.2 (7-14) | 6.9 (5-9) | 12% | KOOS at follow-up | N |
| Dabis (2020) [10] | 20 | Primary repair with internal brace | 12.9 (5-16) | 2.73 (2.4 - 4.3) | 0 % | Lysholm and KOOS pre-op and at follow-up | 100 % |
| Lanzetti (2020) [31] | 42 | P | 12.5 (11-14) | 8 (6 –10.2) | 2 (4.8%) re-ruptures  1 (2.4%) superficial soft tissue infection | Pedi-IKDC and Lysholm pre-op and at follow-up | N |
| Roberti di Sarsina (2019)[10] | 20 | P | 12.3 (8-13) | 4.5 (2.8-10.3) | 0 % | IKDC, Lysholm and KOOS pre-op and at follow-up | N |
| Wilson (2019) [53] | 60 | T* | 13 (11 - 16) | 3.2 (2.0 – 6.5) | 3 (5.3%) re-ruptures | Pedi-IKDC at follow-up | 39 % |
| Chambers (2019) [7] | 24 | P | 12.3 +0.9 | 2.6 +1.5 | 7 (29.2%) revision (hardware removal)  2 (8.3%) re-ruptures | Pedi-IKDC at follow-up | 46 % |
| Sugimoto (2018) [51] | 93 | T+P | PM-HS: 13,6 +1  PF-HS: 13.4 +0.7  PM-ITB: 12.5 +1.3 | 0.5 | 0% | No PROMs | No PROMs |
| Kocher (2018) [28] | 237 | P | 11.2 +1,7 | Physical examination: 2,15  PROM: 6.2 (2.1-24.9) | 9 (6.6%) re-ruptures | Pedi-IKDC and Lysholm at follow-up | 57 % |
| Madelaine (2018)[7] | 53 | N | 11.7 (9.2-14.2) | 3.2 (+2) | Nonoperative | No PROMs | No PROMs |
| Reid (2017) [9] | 100 | T | 14.5 (+ 2.6) | 4 (+ 2.2) | 24%  9 % rerupture | KOOS at follow-up | 80% |
| Faunø (2016) [14] | 39 | T | 11.7 (9-14) | 5.7 (2.4-12.3) | No information | KOOS pre-op and at follow-up | N |
| Domzalski (2016)[1] | 22 | T | Girls: 11.2 (10.6-11.8)  Boys: 12.3 (10.5-13.2) | 6.4 (4.5-8.5) | 0% | IKDC and Lysholm pre-operative and at follow-up | 100% |
| Falciglia (2016) [13] | 33 | P | 12.4 (10-14.2) | A:13,6 (10.8-16.2)  B: 6.3 (3.6 – 9.8) | 2 (6.1%) re-ruptures | IKDC at follow-up | 100 % |
| Calvo (2015) [6] | 27 | T | 13 (12-16) | 10.6 (10-13) | 3 (11.1%) re-ruptures | IKDC and Lysholm pre-op and at follow-up | N |
| Willimon (2015) [12] | 22 | P | 11.8 (9.9-14.0) | 3 (1.0-6.9) | 3 (14%) rerupture | Pedi-IKDC, Lysholm at follow-up | N |
| Moksnes (2013) [42] | 46 | N | 11.0 (7.0 -12.9) | 3.2 +1.1 | Nonoperative treatment | No PROMs | No PROMs |
| Kim (2012) [25] | 25 | T | Boys: 16.3 (9.7- 17.8)  Girls: 16.5 (14.2 – 17.8) | 6.2 (2.1 – 18) | No information | Lysholm and IKDC pre-op and at follow-up | 16 % |
| Courvoisier (2011) [9] | 38 | T | 14 (11.0 – 15.0) | 3.0 (2.0 – 4.0) | 3 (7.9%) re-ruptures  2 (5.3%) because of instability | IKDC at follow-up | 100 % |
| Nikolaou (2011) [43] | 94 | T | 13.7 (11.6-15.9) | 3.2 (2.0-5.0) | 4 (4.3%) re-rupture | Lysholm at follow-up | 100 % |
| Bonnard (2011) [5] | 57 | P | 12.2 (6.8 – 14.5) | 5.5 (2.0 – 14.0) | 3 (5.4%) re-rupture | IKDC at follow-up | 100 % |
| Streich (2010) [50] | 31 | T | 11 (9-12) | 5.8 (3.4-7.1) | 0% | IKDC and Lysholm at follow-up | N |
| Kopf (2010) [30] | 14 | T | 14.4 (11-16) | 7.0 (1.9-11.1) | 0% | KOS-ADLS, Lysholm and IKDC at follow-up | 100 % |
| Henry (2009) [21] | 56 | T | 12.4 (5.0 – 16.8) | 2.3 (1.0 – 6.8) | 2 (3.6%) re-ruptures | IKDC at follow-up | 100 % |
| Cohen (2009) [8] | 26 | T | 13.3 (11-15) | 3.8 (2.0 -7.0) | 3 (11.5%) re-ruptures | IKDC and Lysholm at follow-up | 100 % |
| Marx (2009) [36] | 55 | T | 13.4 (8.4 – 16.6) | 3.2 (1.0 – 7.5) | 1 (5.5%) re-rupture | IKDC, Cincinnati Score and Lysholm at follow-up | 100 % |
| Liddle (2008) [32] | 17 | T | 12.1 (9.5-14.0) | 3.8 (2.1-8.3) | 1 (5.9%) re-rupture | Lysholm pre-op and sat follow-up, IKDC at follow-up | 100 % |
| Moksnes (2008) [41] | 26 | N | 10.3 (5.2-12.7) | 3,8 (2.0-9.0) | Nonoperative | IKDC, KOS-ADLS and Lysholm at follow-up | 77 % |
| Kocher (2007) [29] | 59 | T | 14.7 (11,6-16.9) | 3.6 (2.0-10.2) | 2 (3%) re-ruptures | IKDC and Lysholm at follow-up | 100 % |
| Arbes (2007) [3] | 20 | T | 13.9 (9-15) | 5.4 (0.5 – 10.5) | No information | IKDC and KOOS at follow-up | 100 % |
| Gebhard (2006) [18] | 40 | P | 11.9 (7-14) | 2.8 (1.1 – 17.0) | 3 (8%) re-ruptures | IKDC and Lysholm at follow-up | N |
| Steadman (2006) [49] | 13 | P | 13 (10-16) | 5.8 (2.2 – 9.4) | 3 (23%) re-ruptures | Lysholm at follow-up | 77 % |
| McIntosh (2006) [38] | 16 | T | 13.6 (11.2 – 14.9) | 3.4 (2.0 – 9.3) | 2 (12.5 %) re-ruptures  3 (18.8%) hardware removal | Lysholm and IKDC at follow-up | 100 % |
| Gaulrapp (2006) [17] | 53 | T | 13.9 (9-16) | 6.5 (3.0 – 11.0) | 4 (7,5%) wound healing problems  2 (3.8%) screw fractures or metal implant removal  1 (1.9%) early infection | Lysholm and IKDC at follow-up | 100 % |
| Kocher (2005) [27] | 44 | P | 10.3 (3.6 – 14.0) | 5.3 (2.0 – 15.1) | 2 (4.8%) re-ruptures | Lysholm and IKDC at follow-up | 100 % |
| Seon (2005) [46] | 11 | T | 14.7 (13.1-15.5) | 6.5 (3.8-10.9) | No information | Lysholm pre-op and at follow-up | N |
| Shelbourne (2004) [48] | 16 | T | 14.8 (13.1-15.8) | 3.4 +1.1 | 1 (6.3%) re-rupture | IKDC and Modified Noyes Survey at follow-up | N |
| Anderson (2004) [2] | 12 | P | 13.3 +1.3 | 4.1 (2.0 – 8.1) | No information | IKDC at follow-up | 100 % |
| Woods (2004) [54] | 13 | N | 13.8 (11.0 -16.0) | 5.8 (1.8-24.5) | No information | No PROMs | No PROMs |
| Aichroth (2002) [1] | 45 | T | 13 (11-15) | 4.1 (1.0 – 8.0) | 3 (6.7%) re-ruptures | Lysholm pre-op and at follow-up, IKDC at follow-up | N |
| Fuchs (2002) [16] | 10 | T | 13.2 (9-15) | 3.3 (2.2 – 5.0) | 2 (20%) tibial hardware removal | Lysholm and IKDC at follow-up | 100 % |
| Edwards (2001) [12] | 20 | T | 13.7 (11.8 – 15.6) | 2.8 (1.4 – 7.4) | 2 (10%) re-rupture | Lysholm at follow-up | N |
| Aronowitz (2000) [4] | 21 | T | 13.4 (11.15) | 2.1 (1.0 – 5.0) | 2 (9.5%) hardware removal | Lysholm at follow-up | N |
| Micheli (1999) [39] | 17 | P | 11 (2-14) | 5.5 (2.1 – 14.0) | 0 % | Lysholm at follow-up | 100 % |
| Janarv (1996) [22] | 28 | P | 13.1 (9.9 -15.0) | > 3.0 | 1 (3.6%) re-rupture | Lysholm pre-op and at follow-up for operated and Lysholm at follow-up for non-operatively treated patients | N |
| Mizuta (1995) [40] | 18 | N | 12.8 (10-15) | 4.3 (0.8 – 8.3) | Non-operative | Lysholm at follow-up | 100 % |
| McCarroll (1994) [37] | 60 | T | 13.7 (13-15) | 4.2 (2.0 – 7.0) | 3 (25 %) re-ruptures | No PROMs | No PROMs |
| Graf (1992) [19] | 12 | N | 14.5 (11.7 – 16.3) | > 2.0 | Non-operative | No PROMs | No PROMs |
| Lipscomb (1986) [33] | 24 | P | 13.5 (10-15) | 2.9 (2.0 – 5.0) | No information | No information | N |

Supplementary table: Published outcome studies on the treatment of pediatric anterior cruciate ligament injury. For mean age at inclusion and mean follow-up time range is indicated in brackets and SD as +.

T = Transepifyseal reconstruction, P = Physeal-sparing reconstruction, N = no information. PM-HS = Pediatric males, hamstrings grafts. PF-HS = Pediatric females, hamstring autografts. PM-ITB = Pediatric males with extraphyseal ITB autografts. *Combined transepiphyseal reconstruction and lateral extra-articular tenodesis.

References for supplementary table:

1. Aichroth PM, Patel DV, Zorrilla P (2002) The natural history and treatment of rupture of the anterior cruciate ligament in children and adolescents. A prospective review. J Bone Joint Surg Br 84:38-41

2. Anderson AF (2004) Transepiphyseal replacement of the anterior cruciate ligament using quadruple hamstring grafts in skeletally immature patients. J Bone Joint Surg Am 86-A Suppl 1:201-209

3. Arbes S, Resinger C, Vecsei V, Nau T (2007) The functional outcome of total tears of the anterior cruciate ligament (ACL) in the skeletally immature patient. Int Orthop 31:471-475

4. Aronowitz ER, Ganley TJ, Goode JR, Gregg JR, Meyer JS (2000) Anterior cruciate ligament reconstruction in adolescents with open physes. Am J Sports Med 28:168-175

5. Bonnard C, Fournier J, Babusiaux D, Planchenault M, Bergerault F, de Courtivron B (2011) Physeal-sparing reconstruction of anterior cruciate ligament tears in children: results of 57 cases using patellar tendon. J Bone Joint Surg Br 93:542-547

6. Calvo R, Figueroa D, Gili F, Vaisman A, Mocoçain P, Espinosa M, et al. (2015) Transphyseal anterior cruciate ligament reconstruction in patients with open physes: 10-year follow-up study. Am J Sports Med 43:289-294

7. Chambers CC, Monroe EJ, Allen CR, Pandya NK (2019) Partial Transphyseal Anterior Cruciate Ligament Reconstruction: Clinical, Functional, and Radiographic Outcomes. Am J Sports Med 47:1353-1360

8. Cohen M, Ferretti M, Quarteiro M, Marcondes FB, de Hollanda JP, Amaro JT, et al. (2009) Transphyseal anterior cruciate ligament reconstruction in patients with open physes. Arthroscopy 25:831-838

9. Courvoisier A, Grimaldi M, Plaweski S (2011) Good surgical outcome of transphyseal ACL reconstruction in skeletally immature patients using four-strand hamstring graft. Knee Surg Sports Traumatol Arthrosc 19:588-591

10. Dabis J, Yasen SK, Foster AJ, Pace JL, Wilson AJ (2020) Paediatric proximal ACL tears managed with direct ACL repair is safe, effective and has excellent short-term outcomes. Knee Surg Sports Traumatol Arthrosc 28:2551-2556

11. Domzalski M, Karauda A, Grzegorzewski A, Lebiedzinski R, Zabierek S, Synder M (2016) Anterior Cruciate Ligament Reconstruction Using the Transphyseal Technique in Prepubescent Athletes: Midterm, Prospective Evaluation of Results. Arthroscopy 32:1141-1146

12. Edwards PH, Grana WA (2001) Anterior cruciate ligament reconstruction in the immature athlete: long-term results of intra-articular reconstruction. Am J Knee Surg 14:232-237

13. Falciglia F, Panni AS, Giordano M, Aulisa AG, Guzzanti V (2016) Anterior cruciate ligament reconstruction in adolescents (Tanner stages 2 and 3). Knee Surg Sports Traumatol Arthrosc 24:807-814

14. Faunø P, Rømer L, Nielsen T, Lind M (2016) The Risk of Transphyseal Drilling in Skeletally Immature Patients With Anterior Cruciate Ligament Injury. Orthop J Sports Med 4:2325967116664685

15. Foissey C, Thaunat M, Caron E, Haidar I, Vieira TD, Gomes L, et al. (2022) Combining Anterior Cruciate Ligament Reconstruction With Lateral Extra-Articular Procedures in Skeletally Immature Patients Is Safe and Associated With a Low Failure Rate. Arthrosc Sports Med Rehabil 4:e1941-e1951

16. Fuchs R, Wheatley W, Uribe JW, Hechtman KS, Zvijac JE, Schurhoff MR (2002) Intra-articular anterior cruciate ligament reconstruction using patellar tendon allograft in the skeletally immature patient. Arthroscopy 18:824-828

17. Gaulrapp HM, Haus J (2006) Intraarticular stabilization after anterior cruciate ligament tear in children and adolescents: results 6 years after surgery. Knee Surg Sports Traumatol Arthrosc 14:417-424

18. Gebhard F, Ellermann A, Hoffmann F, Jaeger JH, Friederich NF (2006) Multicenter-study of operative treatment of intraligamentous tears of the anterior cruciate ligament in children and adolescents: comparison of four different techniques. Knee Surg Sports Traumatol Arthrosc 14:797-803

19. Graf BK, Lange RH, Fujisaki CK, Landry GL, Saluja RK (1992) Anterior cruciate ligament tears in skeletally immature patients: meniscal pathology at presentation and after attempted conservative treatment. Arthroscopy 8:229-233

20. Hansson F, Moström EB, Forssblad M, Stålman A, Janarv PM (2022) Long-term evaluation of pediatric ACL reconstruction: high risk of further surgery but a restrictive postoperative management was related to a lower revision rate. Arch Orthop Trauma Surg 142:1951-1961

21. Henry J, Chotel F, Chouteau J, Fessy MH, Berard J, Moyen B (2009) Rupture of the anterior cruciate ligament in children: early reconstruction with open physes or delayed reconstruction to skeletal maturity? Knee Surg Sports Traumatol Arthrosc 17:748-755

22. Janarv PM, Nystrom A, Werner S, Hirsch G (1996) Anterior cruciate ligament injuries in skeletally immature patients. J Pediatr Orthop 16:673-677

23. Kamei G, Nakamae A, Nakata K, Nekomoto A, Tsuji S, Hashiguchi N, et al. (2023) Comparison of clinical outcomes between anterior cruciate ligament reconstruction with over-the-top route procedure and anatomic single-bundle reconstruction in pediatric patients. J Pediatr Orthop B 32:178-184

24. Kew ME, Bodkin S, Diduch DR, Brockmeier SF, Lesevic M, Hart JM, et al. (2022) Reinjury Rates in Adolescent Patients 2 Years Following ACL Reconstruction. J Pediatr Orthop 42:90-95

25. Kim SJ, Shim DW, Park KW (2012) Functional outcome of transphyseal reconstruction of the anterior cruciate ligament in skeletally immature patients. Knee Surg Relat Res 24:173-179

26. Kitchen BT, Mitchell BC, Cognetti DJ, Siow MY, Howard R, Carroll AN, et al. (2022) Outcomes After Hamstring ACL Reconstruction With Suture Tape Reinforcement in Adolescent Athletes. Orthop J Sports Med 10:23259671221085577

27. Kocher MS, Garg S, Micheli LJ (2005) Physeal sparing reconstruction of the anterior cruciate ligament in skeletally immature prepubescent children and adolescents. J Bone Joint Surg Am 87:2371-2379

28. Kocher MS, Heyworth BE, Fabricant PD, Tepolt FA, Micheli LJ (2018) Outcomes of Physeal-Sparing ACL Reconstruction with Iliotibial Band Autograft in Skeletally Immature Prepubescent Children. J Bone Joint Surg Am 100:1087-1094

29. Kocher MS, Smith JT, Zoric BJ, Lee B, Micheli LJ (2007) Transphyseal anterior cruciate ligament reconstruction in skeletally immature pubescent adolescents. J Bone Joint Surg Am 89:2632-2639

30. Kopf S, Schenkengel JP, Wieners G, Stärke C, Becker R (2010) No bone tunnel enlargement in patients with open growth plates after transphyseal ACL reconstruction. Knee Surg Sports Traumatol Arthrosc 18:1445-1451

31. Lanzetti RM, Pace V, Ciompi A, Perugia D, Spoliti M, Falez F, et al. (2020) Over the top anterior cruciate ligament reconstruction in patients with open physes: a long-term follow-up study. Int Orthop 44:771-778

32. Liddle AD, Imbuldeniya AM, Hunt DM (2008) Transphyseal reconstruction of the anterior cruciate ligament in prepubescent children. J Bone Joint Surg Br 90:1317-1322

33. Lipscomb AB, Anderson AF (1986) Tears of the anterior cruciate ligament in adolescents. J Bone Joint Surg Am 68:19-28

34. Madelaine A, Fournier G, Sappey-Marinier E, Madelaine T, Seil R, Lefevre N, et al. (2018) Conservative management of anterior cruciate ligament injury in paediatric population: About 53 patients. Orthop Traumatol Surg Res 104:S169-s173

35. Maheshwer B, Polce EM, Parvaresh KC, Paul K, Yanke AB, Forsythe B, et al. (2022) Establishing Clinically Significant Outcomes After Anterior Cruciate Ligament Reconstruction in Pediatric Patients. J Pediatr Orthop 42:e641-e648

36. Marx A, Siebold R, Sobau C, Saxler G, Ellermann A (2008) [ACL reconstruction in skeletally immature patients]. Z Orthop Unfall 146:715-719

37. McCarroll JR, Shelbourne KD, Porter DA, Rettig AC, Murray S (1994) Patellar tendon graft reconstruction for midsubstance anterior cruciate ligament rupture in junior high school athletes. An algorithm for management. Am J Sports Med 22:478-484

38. McIntosh AL, Dahm DL, Stuart MJ (2006) Anterior cruciate ligament reconstruction in the skeletally immature patient. Arthroscopy 22:1325-1330

39. Micheli LJ, Metzl JD, Di Canzio J, Zurakowski D (1999) Anterior cruciate ligament reconstructive surgery in adolescent soccer and basketball players. Clin J Sport Med 9:138-141

40. Mizuta H, Kubota K, Shiraishi M, Otsuka Y, Nagamoto N, Takagi K (1995) The conservative treatment of complete tears of the anterior cruciate ligament in skeletally immature patients. J Bone Joint Surg Br 77:890-894

41. Moksnes H, Engebretsen L, Risberg MA (2008) Performance-based functional outcome for children 12 years or younger following anterior cruciate ligament injury: a two to nine-year follow-up study. Knee Surg Sports Traumatol Arthrosc 16:214-223

42. Moksnes H, Engebretsen L, Risberg MA (2013) Prevalence and incidence of new meniscus and cartilage injuries after a nonoperative treatment algorithm for ACL tears in skeletally immature children: a prospective MRI study. Am J Sports Med 41:1771-1779

43. Nikolaou P, Kalliakmanis A, Bousgas D, Zourntos S (2011) Intraarticular stabilization following anterior cruciate ligament injury in children and adolescents. Knee Surg Sports Traumatol Arthrosc 19:801-805

44. Reid D, Leigh W, Wilkins S, Willis R, Twaddle B, Walsh S (2017) A 10-year Retrospective Review of Functional Outcomes of Adolescent Anterior Cruciate Ligament Reconstruction. J Pediatr Orthop 37:133-137

45. Roberti di Sarsina T, Macchiarola L, Signorelli C, Grassi A, Raggi F, Marcheggiani Muccioli GM, et al. (2019) Anterior cruciate ligament reconstruction with an all-epiphyseal "over-the-top" technique is safe and shows low rate of failure in skeletally immature athletes. Knee Surg Sports Traumatol Arthrosc 27:498-506

46. Seon JK, Song EK, Yoon TR, Park SJ (2005) Transphyseal reconstruction of the anterior cruciate ligament using hamstring autograft in skeletally immature adolescents. J Korean Med Sci 20:1034-1038

47. Shamrock AG, Duchman KR, Cates WT, Cates RA, Khazi ZM, Westermann RW, et al. (2022) Outcomes Following Primary Anterior Cruciate Ligament Reconstruction Using a Partial Transphyseal (Over-the-Top) Technique in Skeletally Immature Patients. Iowa Orthop J 42:179-186

48. Shelbourne KD, Gray T, Wiley BV (2004) Results of transphyseal anterior cruciate ligament reconstruction using patellar tendon autograft in tanner stage 3 or 4 adolescents with clearly open growth plates. Am J Sports Med 32:1218-1222

49. Steadman JR, Cameron-Donaldson ML, Briggs KK, Rodkey WG (2006) A minimally invasive technique ("healing response") to treat proximal ACL injuries in skeletally immature athletes. J Knee Surg 19:8-13

50. Streich NA, Barie A, Gotterbarm T, Keil M, Schmitt H (2010) Transphyseal reconstruction of the anterior cruciate ligament in prepubescent athletes. Knee Surg Sports Traumatol Arthrosc 18:1481-1486

51. Sugimoto D, Heyworth BE, Collins SE, Fallon RT, Kocher MS, Micheli LJ (2018) Comparison of Lower Extremity Recovery After Anterior Cruciate Ligament Reconstruction With Transphyseal Hamstring Versus Extraphyseal Iliotibial Band Techniques in Skeletally Immature Athletes. Orthop J Sports Med 6:2325967118768044

52. Willimon SC, Jones CR, Herzog MM, May KH, Leake MJ, Busch MT (2015) Micheli Anterior Cruciate Ligament Reconstruction in Skeletally Immature Youths: A Retrospective Case Series With a Mean 3-Year Follow-up. Am J Sports Med 43:2974-2981

53. Wilson PL, Wyatt CW, Wagner KJ, 3rd, Boes N, Sabatino MJ, Ellis HB, Jr. (2019) Combined Transphyseal and Lateral Extra-articular Pediatric Anterior Cruciate Ligament Reconstruction: A Novel Technique to Reduce ACL Reinjury While Allowing for Growth. Am J Sports Med 47:3356-3364

54. Woods GW, O'Connor DP (2004) Delayed anterior cruciate ligament reconstruction in adolescents with open physes. Am J Sports Med 32:201-210

55. Zhang L, Liang Q, Zhao Z, Zhang L, Kang X, Tian B, et al. (2023) Robot-assisted all-epiphyseal anterior cruciate ligament reconstruction in skeletally immature patients: a retrospective study. Int Orthop 47:429-435

56. Zimmerer A, Schneider MM, Semann C, Schopf W, Sobau C, Ellermann A (2021) 17-Year Results following Transepiphyseal Anterior Cruciate Ligament Reconstruction in Children and Adolescents. Z Orthop Unfall;10.1055/a-1352-5541
